# Time spent playing video games is unlikely to impact well-being

**DOI:** 10.1098/rsos.220411

**Published:** 2022-07-27

**Authors:** Matti Vuorre, Niklas Johannes, Kristoffer Magnusson, Andrew K. Przybylski

**Affiliations:** ^1^ Oxford Internet Institute, University of Oxford, Oxford, Oxfordshire OX1 3JS, UK; ^2^ Centre for Psychiatry Research, Department of Clinical Neuroscience, Karolinska Institutet, and Stockholm Health Care Services, Region Stockholm, Stockholm, Sweden

**Keywords:** video games, well-being, play behaviour, human motivation

## Abstract

Video games are a massively popular form of entertainment, socializing, cooperation and competition. Games' ubiquity fuels fears that they cause poor mental health, and major health bodies and national governments have made far-reaching policy decisions to address games’ potential risks, despite lacking adequate supporting data. The concern–evidence mismatch underscores that we know too little about games' impacts on well-being. We addressed this disconnect by linking six weeks of 38 935 players’ objective game-behaviour data, provided by seven global game publishers, with three waves of their self-reported well-being that we collected. We found little to no evidence for a causal connection between game play and well-being. However, results suggested that motivations play a role in players' well-being. For good or ill, the average effects of time spent playing video games on players’ well-being are probably very small, and further industry data are required to determine potential risks and supportive factors to health.

## 1. Introduction

Billions of people play video games [1] and their popularity has led stakeholders to express concerns, but also hopes, about their effects on players. Many have warned about video games' possible addictive qualities and their potential harm to players’ well-being (e.g. [2,3]), leading to far-reaching and widely contested health policy decisions [4,5], such as China limiting young people's game play to one hour per day [6]. Conversely, video games may help players relax and recharge [7–9], and even serve as psychological treatment [10]. Consequently, games have the potential to affect well-being on a global scale. It is therefore critical that researchers provide robust, credible and relevant evidence to inform policymakers [11,12]. However, current evidence does not meet these criteria and tells us little about the causal links between video games and well-being [13,14].

Why is current evidence inadequate? On the theoretical level, there are vastly different approaches to investigating the effects of games. Researchers studying the psychology of play highlight that games can help players try out different social roles, experience power in a safe environment, or experience a state of flow [15–17]. Earlier research traditions invoked the displacement hypothesis to explain the effects of media, taking for granted the idea that engaging with media displaces face-to-face interaction, leading to lower well-being [18]. However, recent studies find little to no evidence for the hypothesis [19,20]. By contrast, research based on theories of human motivation indicates the reasons why people use technology (i.e. quality of behaviour) is more relevant to their well-being than the amount of time they spend engaged (i.e. quantity of behaviour). The extent to which play is enjoyable and intrinsically motivated rather than extrinsically motivated is a consistent predictor of players' well-being [9,21–23]. Taken together, these different approaches to studying games, and mixed findings therein, have made it challenging to deliver actionable evidence to stakeholders. This lack of consistent evidence should give pause to researchers trying to deliver advice to health policymakers [12].

Compounding these theoretical challenges, four notable methodological weaknesses limit current evidence quality. First, in typical studies, participants complete experiments designed to imitate play [24]. Such experimental manipulations of game play facilitate causal inference but do not accurately capture play as it occurs naturally [25]. Second, naturalistic studies of game play have almost exclusively relied on retrospective self-reports of play, which are inaccurate indicators of actual behaviour [26–28]. Third, even where studies have used accurate data, these are cross-sectional and do not adequately address potential *causal* effects of games on well-being [21]. Fourth, investigations have typically been limited to a handful of games, which hampers researchers' ability to generalize their findings to talk about games in general [29].

The present study addresses these limitations by studying naturalistic play in a sample of 38 935 players; collaborating with game publishers to obtain accurate measures of objective behaviour; collecting three waves of longitudinal data on player well-being and motivations; and examining seven popular video game titles rather than focusing on a single title, genre, or type of player. Our first, and primary, objective was to estimate the causal effects of video game play on well-being: the extent to which the quantity of play—the average daily hours played over a two-week period—impacts players’ well-being. We had two secondary objectives. There is a long tradition in media effects research to see people as active in choosing media to, for example, regulate how they feel [30–32]. Therefore, our second objective was to estimate the reciprocal effect: the extent to which well-being affects subsequent video game play [19,33]. In a similar vein, we should conceive of video game players as actively experiencing play—not as passive entities to whom play and its effects just ‘happen’ [34,35]. Much research has shown the importance of considering how people experience video game play: an intrinsic, self-determined experience will differ from feeling externally pressured to play [21,23]. Therefore, our third objective was to investigate how the motivational dimensions of play (intrinsic or extrinsic) related to shifts in well-being.

## 2. Methods

### 2.1. Participants and procedure

We collaborated with game publishers who recruited players with emails to participate in a three-wave panel study. Seven publishers participated with the following games: *Animal Crossing: New Horizons* (Nintendo of America; *N* = 13 646), *Apex Legends* (Electronic Arts; *N* = 1158), *Eve Online* (CCP Games; *N* = 905), *Forza Horizon 4* (Microsoft; *N* = 1981), *Gran Turismo Sport* (Sony Interactive Entertainment; *N* = 19 258), *Outriders* (Square Enix; *N* = 1530) and *The Crew 2* (Ubisoft; *N* = 457). The emails targeted the general English-speaking player bases of these publishers in Australia, Canada, India, Ireland, New Zealand, South Africa, United Kingdom and the United States. Publishers invited active players of the selected game to participate. Active play was defined as having played the respective game in the past two weeks to two months; variability in this interval between publishers was due to differences in how many players regularly played a given game, so that an adequate sample could be invited.

We did not plan to recruit a specific number of participants, but instead aimed to collect the largest possible sample of players from each participating game publisher, given their player base sizes and capabilities of contacting them. The invitation email campaigns were carried out from May to September in 2021, with different publishers inviting between 30 000 and 1 729 627 players. The emails had short invitations to the study and contained a link to our survey hosted at our institution. Upon entering the survey, participants first read information on the purposes and procedures of the study, were given the option to consent, and then proceeded to the survey measures. We counted anyone who consented to the study and responded to at least one survey item as a participant, but participants could leave responses blank or quit or withdraw at any time. Therefore, the sample sizes per analysis vary because of this variable missingness.

The publishers then recontacted all players who participated at the first wave two and four weeks later with invitations to participate in the following waves of the study. Because of the technical implementations of the email campaigns and late responding, the intervals between responses were not exactly two weeks but instead varied slightly (IQR = [13.7, 15.9 days]). We did not implement a response window, but participants could complete the surveys at any time suitable for them.

Initial response rates ranged from 0.1% to 3.0%, in line with a previous study that used similar recruitment methods [21], but retention rates were greater for the following waves (21.2% to 92.1% between games and waves; table 1). At the conclusion of the study, the publishers sent the behavioural data of all consented participants to our team for joining with survey data for analysis. At no point did we collect personally identifiable data or risked participants' privacy. Instead, the survey URL captured a hashed ID per player. The game publishers created those hashed IDs for each player account. We used these hashed, anonymous IDs to connect each player's survey responses to their behavioural data.

**Table 1.** Numbers of players invited to the study, and at each wave. Note: numbers in parentheses are retention rates from the previous wave (or percentages of individuals who participated from everyone who was invited, in Wave 1).

Game	Invites	Wave 1	Wave 2	Wave 3
AC:NH	640 000	13 536 (2.1%)	5049 (37.3%)	4084 (80.9%)
Apex Legends	900 000	1128 (0.1%)	406 (36.0%)	228 (56.2%)
EVE Online	30 000	899 (3.0%)	240 (26.7%)	221 (92.1%)
Forza Horizon 4	834 515	1959 (0.2%)	772 (39.4%)	597 (77.3%)
GT Sport	1 729 677	19 073 (1.1%)	7699 (40.4%)	5512 (71.6%)
Outriders	90 000	1525 (1.7%)	379 (24.9%)	370 (97.6%)
The Crew 2	1 013 000	457 (0.0%)	97 (21.2%)	85 (87.6%)

We were interested in studying active players, defined as playing during the study period. Of the 108 880 consented players, 85% responded to at least one well-being survey item; of those, 42% had played during the study, leading to a final sample of 38 935 active players. Of these players, 77% identified as male, 21% as female, 1.8% as third or non-binary gender, with other responses missing or declined. The participants' median age was 34 years (interquartile range (IQR) = [25, 42]) and they had a reported median 23 years (IQR = [16, 30]) of experience playing games.

The study procedures were granted ethical approval by our institute's Central University Research Ethics Committee (SSH_OII_CIA_21_011). All participants provided informed consent to participate in the study and to have their game-play behaviour data provided by the respective game publisher and reported being 18 years or older.

### 2.2. Measures

#### 2.2.1. Video game behaviour

Participating publishers provided us with each participant's game-play data from two weeks prior to the first wave up to the third wave. Different publishers had access to data at different levels of detail, but all of them provided the start and end times (i.e. durations) of each game session. However, due to differences in the games and logging systems, each game's session start and end times were measured slightly differently because different events could count as session start and end indicators. A participant could have multiple game sessions over the two-week period for each wave. We, therefore, added their session durations to obtain total game time in the two weeks preceding each survey wave. In doing so we excluded all sessions with durations below 0 (0.5%) or above 10 h (1.9%), as extreme values can sometimes happen due to logging errors. In the analyses below, we used the average number of hours per day in the two-week period.

Some games included additional behavioural data, such as information on in-game achievement and social aspects, but we do not report on those variables here.

#### 2.2.2. Well-being

In line with a previous study [21], we queried participants' affective well-being with the scale of positive and negative experiences (SPANE) [36]. Participants reflected on how they had been feeling in the past two weeks, and reported how often they experienced six each of positive (e.g. Pleasant) and negative feelings (e.g. Unpleasant) on a scale from 1 (*Very rarely or never*) to 7 (*Very often or always*). As recommended, we subtracted the mean of negative items from the mean of positive item to form a global measure of affective well-being [36].

We also measured participants' general life satisfaction with the Cantril self-anchoring scale. This one-item measure asked participants to ‘Please imagine a ladder with steps numbered from 0 at the bottom to 10 at the top. The top of the ladder represents the best possible life for you, and the bottom of the ladder represents the worst possible life for you. On which step of the ladder would you say you personally feel you stood over the **past two weeks**?’. Participants responded on a scale from 0 to 10 [37,38].

#### 2.2.3. Motivational experiences

We also measured players' experiences and motivations with the player experience and need satisfaction scale (PENS) [23,39]. This scale asked participants to reflect on their past two weeks of playing the game title, and reporting on their experienced sense of autonomy, competence, relatedness (only when they reported having played with others), intrinsic motivation and extrinsic motivation on subscales with 3–4 items. Participants responded on a scale from 1 (*Strongly disagree*) to 7 (*Strongly agree*). For the analyses reported below, we created indicators for intrinsic and extrinsic motivations by taking the means of the respective subscales.

In addition, participants were asked to estimate their time spent playing the specific title in the past two weeks in hours and minutes.

### 2.3. Data analysis

This study was designed to take the first steps toward identifying the real-world causal impacts of game play on well-being over time. The longitudinal design required us to specify a temporal lag for the hypothetical causal effect. We chose a two-week window for several reasons. Theoretically, video game effects in the short term should accumulate over a short time frame if such effects are indeed consequential, and not transient and immediately dissipating. Practically, the lag needed to be large enough to capture enough play behaviour. If people play games occasionally, a one-day lag might miss most of their play. Last, we decided to measure affective well-being and life satisfaction. Affective well-being is often measured in the short term, whereas life satisfaction is often measured in the long term. A two-week window allowed us to measure both with high fidelity without risking making the measure awkward for participants.

Therefore, our study was designed to detect effects of video game play on well-being that accumulated over a two-week window, such that we would be able to detect these effects in the following two weeks. Our study would miss effects if they were transient, happened immediately but did not accumulate, or happened within the two-week window, but subsided before the following two weeks [40]. Following this reasoning, we identified the following as the most plausible causal pathway: the cross-lagged association between game play in the two weeks prior to the period addressed by the survey questions, e.g. game play during weeks (0, 2] affecting well-being at weeks (2, 4].

To best estimate this effect over time, we specified a bivariate random intercepts cross-lagged panel model using average hours played per day and the well-being scores as the target variables (RICLPM [41,42]). We determined this model as most appropriate because it separates stable traits and their correlations from within-person differences over time, the main inferential target of our study [43]. We estimated the RICLPM separately for affect and life satisfaction, and for each game. We constrained the (cross-)lagged paths to be equal across the two intervals because there is no *a priori* reason to expect their variance over this time period. We used the *lavaan* R package for estimating the game-specific RICLPMs, and treated missingness with full information maximum-likelihood methods [44].

Our investigation was not about the specific seven games in our sample; instead, we considered them a sample from a broader universe of games. Therefore, we summarized the RICLPM parameters across games with Bayesian random-effects meta-analyses. We focused on unstandardized parameters because they facilitate understanding magnitudes on the variables' natural scales ([45]; see electronic supplementary material (ESM) for the corresponding standardized parameters). We chose to use probabilistic inference for the meta-analysis because the sample of games was somewhat small, and it allowed us to incorporate vaguely informative prior information: We assumed a Student's *t* distribution with seven degrees of freedom, mean of 0 and standard deviation of 0.25 on the standard deviation between games. This prior distribution reflects our view that games are likely to have different associations, but that very large differences between games are unlikely (for example, this prior allocates approximately 35% probability for differences greater than 0.25, and approximately 9% probability for differences greater than 0.5).

In the analyses reported here, the sample sizes vary between analyses/variables due to differential data missingness. We used R for all data analyses [46].

#### 2.3.1. On causality

**Figure 1.**
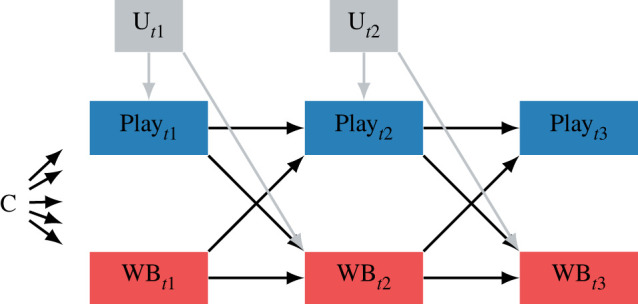
Illustration of the causal model addressed by the current study, where play and well-being can have reciprocal effects on each other over time. The RICLPM can deal with C (time-invariant unknown covariates that affect play and WB (well-being)), but not an unknown time-varying variable (U) that confounds the effect of play on well-being. *t*1 = first time point.

Given this modelling strategy, the causal implications of our results will rest on assuming no time-varying confounders, no selection bias and the correct time lag [47]. However, we are unable to control for variables that might create spurious relations between game time and well-being [48–50], as we did not collect data on these variables, nor was there strong theory to identify such confounders. To clarify, figure 1 depicts how an unknown time-varying confounder (U) could bias the effect. For example, how much leisure time people have might increase both their play now and their well-being later. This would bias a true negative effect toward the null. Confounding may be even more complex: more leisure time could cause more boredom, which increases play time, but decreases well-being, and thereby bias a true positive effect toward the null. In addition, it is possible that non-additive effects of time-invariant confounders—such as interactions with time-varying variables—could bias the observed effects (e.g. [51]).

Another closely related issue is self-selection. We observed low response rates and notable attrition. It is probable that self-selection might bias the estimates presented below. For example, older and more experienced players could be more passionate about research and could plausibly have figured out ways to fit games into their lives so playing will not negatively impact well-being. Under this self-selection, a true negative effect would be biased toward the null. Similarly, dropout between waves could be caused by unobserved variables, violating the missing at random assumption. For example, missingness could relate to lower well-being and more play, masking a negative effect. We return to issues related to attrition after presenting our results.

Last, extending previous work [21], we chose a two-week lag to capture enough variation in play. However, any actual effects may be too short-lived to be detected with our design, or accumulate over longer periods of time [52]. Thus, the potential causal effect discussed in this paper specifically refers to an effect carried over two weeks of play on the subsequent two weeks of well-being. Future work must investigate the temporal properties of potential effects: studies with both a higher measurement resolution and a longer overall time span are needed to detect potential more transient or slower effects, respectively. Given the difficulties in attempting to infer causality from observational data, we are nevertheless specific that our study aims to address the *causal effects* of game play, however tentatively, and hope that this clarity helps the interpretation of our results, and design of future studies [53].

### 2.4. Data, code and materials

The data, annotated code required to process and analyse them, supplementary analyses, and survey materials are available at https://osf.io/fb38n/ (henceforth referred to as electronic supplementary material, ESM). That page also includes details on data processing, such as how session durations were cleaned, exact item wordings, complete surveys and additional data not analysed here. This study was not preregistered.

## 3. Results

**Figure 2.**
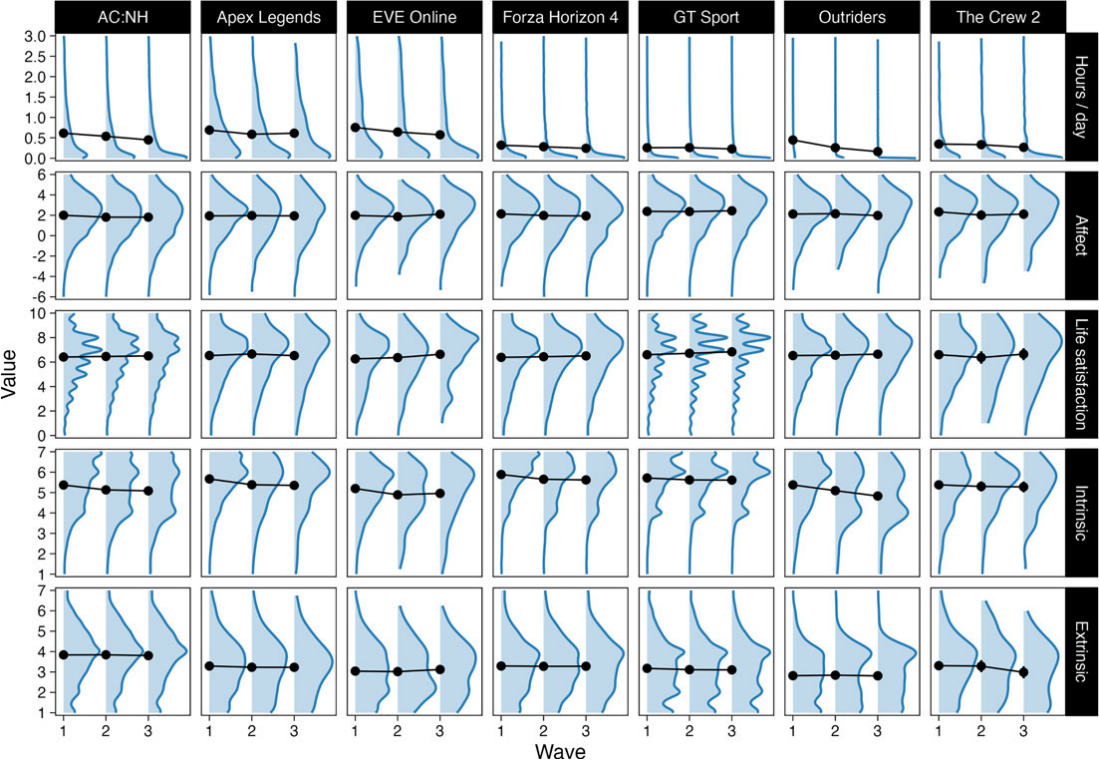
Distributions of key variables (rows) over time and by game (columns). Points and lines are means at each wave. We excluded hours of play per day over 3 (2.6%) from this figure for clarity.

The amount of time played was variable across the games and showed a small decrease on average over the three waves, which was probably attributable to differential attrition (figure 2, top panels; *b*_wave_ = −0.09, [−0.15, −0.04]; estimates from a generalized linear mixed model with random intercepts for players, and random intercepts and slopes for games). Players' affective well-being decreased slightly (*b*_wave_ = −0.05, [−0.10, −0.01]) and life satisfaction slightly increased (*b*_wave_ = 0.05, [0.03, 0.08]) over the six weeks of the study. Players’ intrinsic (*b*_wave_ = −0.18, [−0.23, −0.13]) and extrinsic motivations (*b*_wave_ = −0.01, [−0.03, 0.01]) also slightly decreased over time (figure 2). These patterns suggest that missingness was not completely random; participation was probably driven by players' well-being and motivations (see section on attrition).

### 3.1. Effects between play and well-being over time

**Figure 3.**
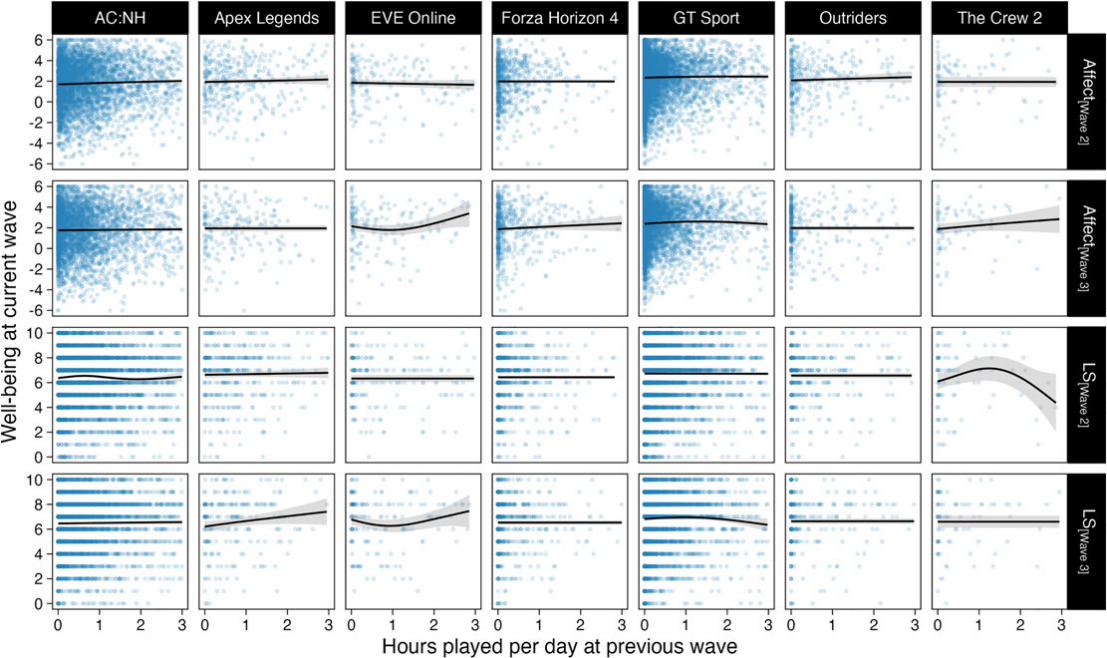
Scatterplots of well-being scores and hours played at the previous wave. Points are individual participants' data, and lines and shades are model fits and 95% CIs from exploratory generalized additive models with penalized cubic splines. The *x*-axis is truncated at 3 hours per day for this figure for clarity.

**Figure 4.**
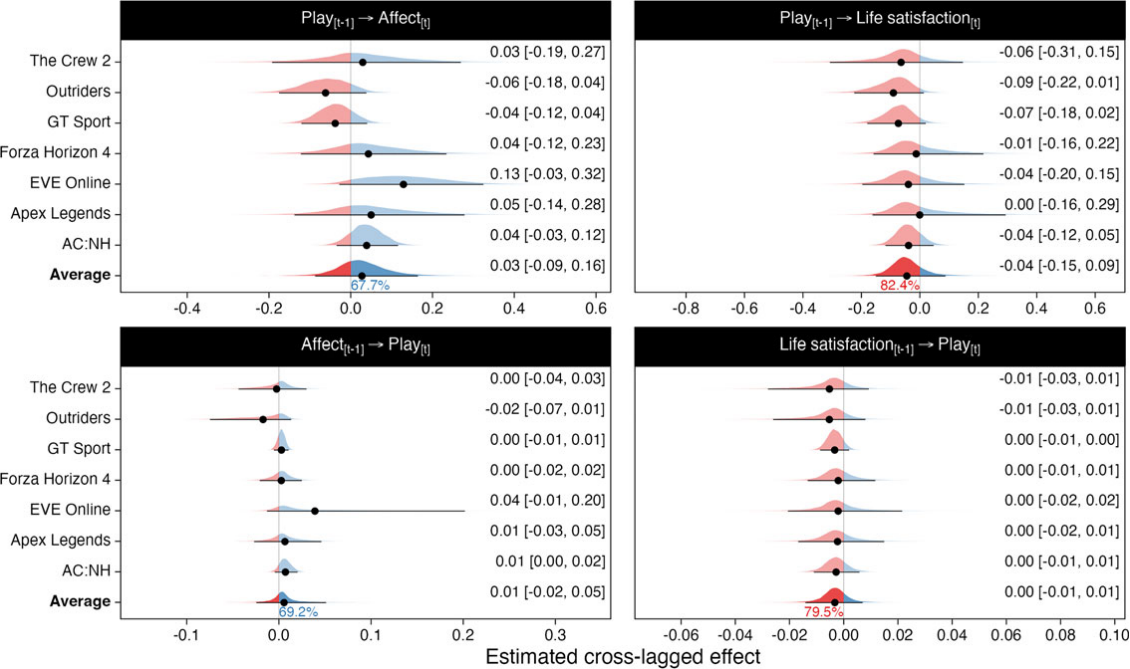
Random-effects meta-analytic estimates of the unstandardized cross-lagged effects of average daily hours played on well-being (top left: on affect; top right: on life satisfaction), and well-being on average daily play time (bottom left: affect's effect on play time; bottom right: life satisfaction's effect on play time). Shaded areas indicate parameters' approximate posterior densities. Percentages below the average parameters indicate posterior probabilities of the effects' directions. The points and intervals indicate posterior means and 95% credibility intervals (CI; reproduced in text on the right).

We then focused on our first research objective: determining the extent to which game play affects well-being. Scatterplots describing the associations between (lagged) hours played and well-being are shown in figure 3. The meta-analysis of play time and affect indicated that, on average, video game play had little to no effect on affect, with 68% posterior probability of a positive effect (figure 4, top left). The 95% most likely effect sizes of a one-hour daily increase in play on the 13-point SPANE scale ([−0.09, 0.16]) indicated that the effect was not credibly different from zero: the magnitude and associated uncertainty of this effect suggests that there is little to no practical causal connection (given our assumptions described above) between game play in the preceding two weeks and current affect.

The meta-analysis of play time and life satisfaction indicated similar results: increasing one's play by one hour per day would lead to a −0.04 [−0.15, 0.09] point change in the 11-point life satisfaction scale (figure 4, top right). The posterior probability for a negative effect was 82%.

To put these numbers in perspective, the average range of changes in players' average weekly hours of play in this sample was 0.48 h. Thus, if an average player went from their minimum amount of play to the greatest, their affect would change by 0.013 [−0.04, 0.08] units on the SPANE scale, and their life satisfaction by −0.02 [−0.07, 0.04]: on average, the amount of time a person spends playing games is unlikely to have a meaningful impact on their well-being.

The above numbers indicated results for the average game, but individual games’ results are also summarized in figure 4. Considering our games a random sample from the broader universe of games, the meta-analytic models' across-game standard deviations (affect: 0.11 [0.01, 0.27]; life satisfaction: 0.08 [0.00, 0.26]) indicate that individual games are likely to differ from one another in their potential effects.

We then turned to our second research objective: investigating the reciprocal effects of affect (figure 4, bottom left) and life satisfaction (figure 4, bottom right) on play. The average meta-analytic estimate across games indicated that a one-unit increase on the 13-point SPANE affect scale would lead to a 0.01 [−0.02, 0.05] hour per week change in play. Similarly, life satisfaction had a practically zero effect on game play (0.00 [−0.01, 0.01]). This suggests little to no practical causal connection linking well-being to subsequent game play.

The RICLPM included other parameters of subsidiary interest. First, the autocorrelation parameters indicated that affect and life satisfaction were modest predictors of themselves (*b*_affect[*t* − 1]_ = 0.40 [0.29, 0.53]; *b*_life satisfaction[*t* − 1]_ = 0.20 [0.12, 0.31]). Second, the covariances of the random intercepts, indicating the extent to which people who tend to play more are also more likely to report higher well-being, were not credibly different from zero for either affect (meta-analysed covariance: 0.02 [−0.13, 0.15]) or life satisfaction (0.03 [−0.07, 0.14]). Third, the meta-analytic average estimates of the within-person game-play–well-being covariances at time 1 were not credibly different from zero (0.04 [−0.01, 0.09] (affect), 0.00 [−0.06, 0.06] (life satisfaction), see ESM for game-specific results), suggesting that there was little simultaneous association between the two.

We then considered several control analyses addressing the association between hours spent playing and well-being. First, our causal model was premised on the idea that game play affects well-being in the subsequent two-week window. It is possible that, in addition to a lagged effect, play affects well-being simultaneously or with a much shorter time-lag. In that case, the RICLPM might adjust away a cross-lagged effect of interest by also including the autoregressive parameters. To test this possibility, we examined a variant of the RICLPM that did not include autoregressions. The key meta-analytic average cross-lagged parameters in these models were nearly identical to our main analysis (0.04 [−0.05, 0.15] (affect) and −0.04 [−0.14, 0.06] (life satisfaction), see ESM for game-specific estimates), suggesting that our conclusions are robust to this assumption.

Second, positive and negative affect are often considered as separate, independent components of affective well-being, and it is possible that game play affects them differently. We therefore also conducted the above analyses but on these SPANE subscales. The meta-analytic average cross-lagged parameter of play on negative affect was −0.03 [−0.10, 0.02], and 0.00 [−0.08, 0.08] on positive affect. These results closely mirrored the ones from the overall SPANE scale described above (see SOM for full results.)

In sum, analyses testing our first two objectives provided little evidence in favour of the idea that shifts in time spent playing games, on average, have negative or positive effects on players’ well-being, or that well-being directly affects how much time individuals spend playing games.

#### 3.1.1. Role of motivational experiences in well-being

**Figure 5.**
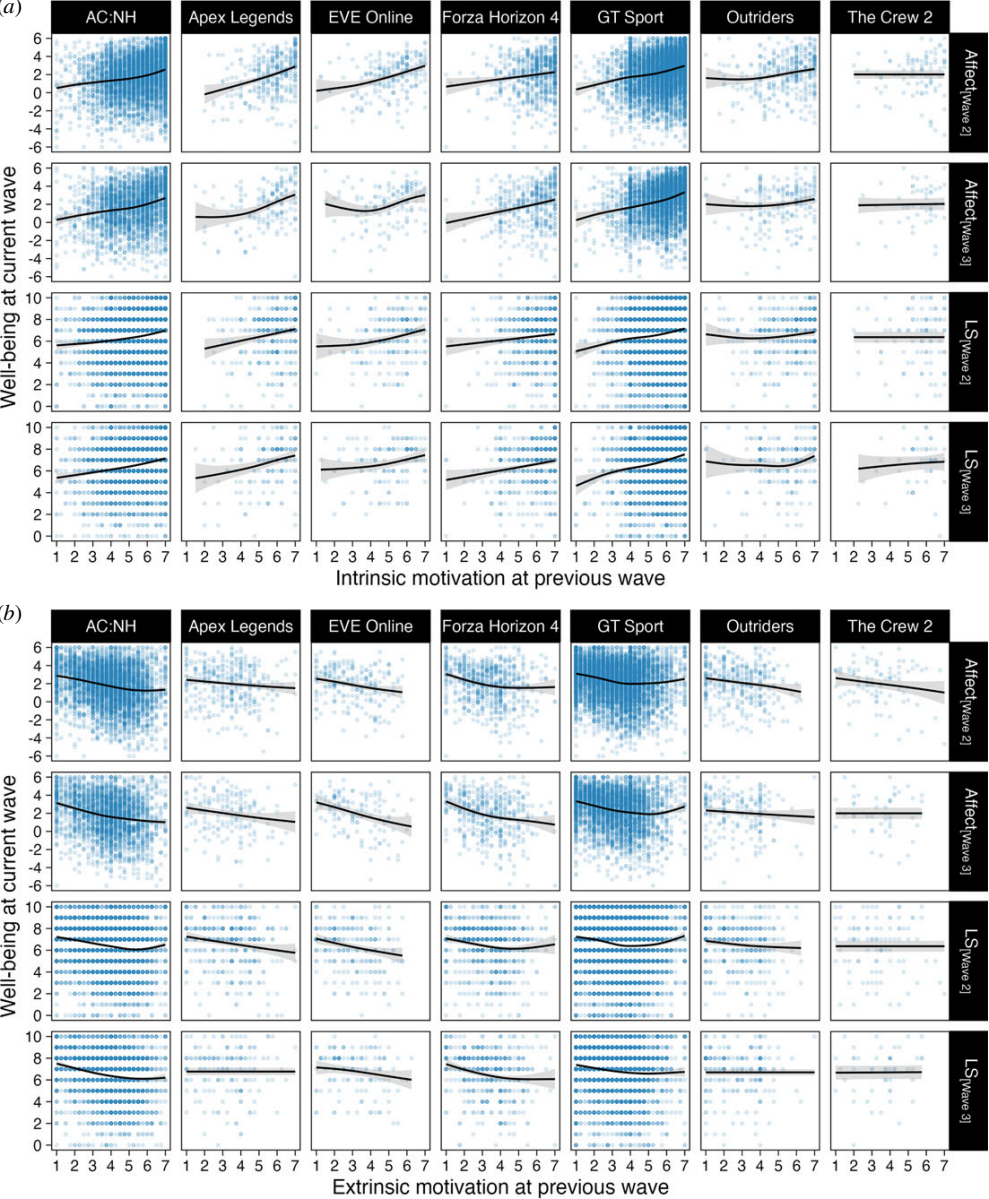
Scatterplots of well-being scores on intrinsic (*a*) and extrinsic (*b*) motivation in the previous wave. Points are individual participants' data, and lines and shades are model fits and 95 %CIs from exploratory generalized additive models with penalized cubic splines.

We then turned to our third research objective: investigating the roles of motivational experiences during play in players' subsequent well-being. We conducted additional RICLP models and used intrinsic and extrinsic motivation scores (subscale means) in place of play time in the model with well-being scores. These data are shown in figure 5.

**Figure 6.**
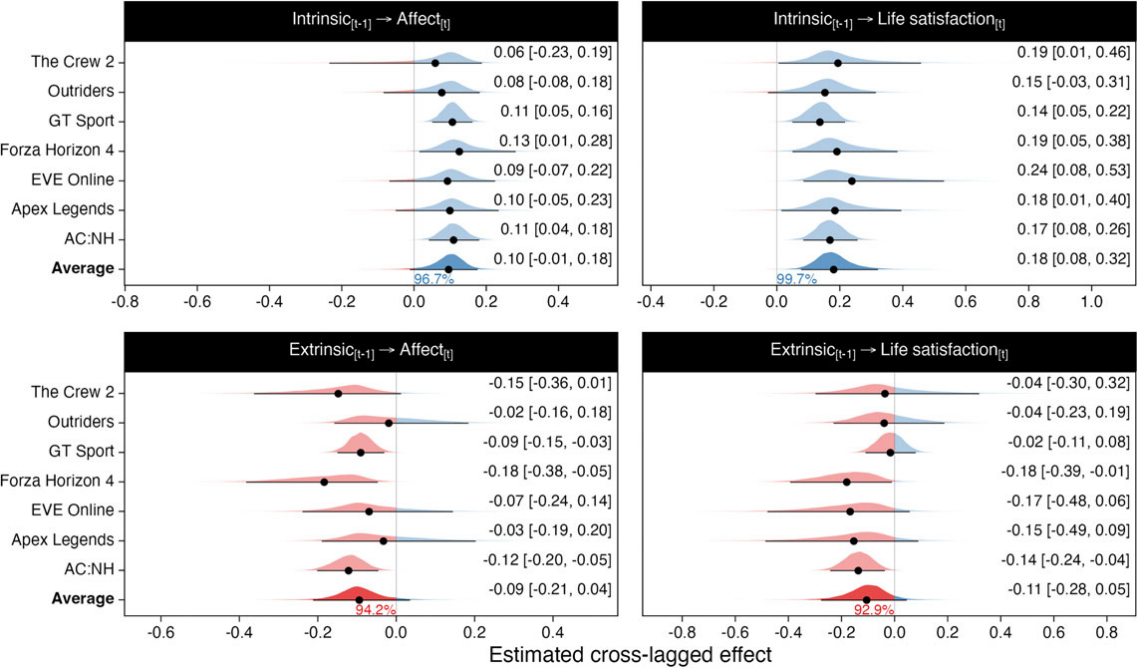
Random-effects meta-analytic estimates of the unstandardized cross-lagged effects of intrinsic motivation (top) and extrinsic motivation (bottom row) on well-being.

We ran four models: affect predicted by each motivation, and life satisfaction predicted by each motivation. As above, we constrained the lagged and cross-lagged coefficients across waves. After conducting these models separately for each game, we then aggregated the results with random-effects meta-analyses as above. The results of these analyses are presented in figure 6.

First, there was an average positive effect of intrinsic motivation on both affect and life satisfaction, with posterior probabilities in excess of 96% in favour of a positive effect (figure 6, top row). These effects were consistent across games in both magnitude and direction and indicated that on average a one-unit increase on the intrinsic motivation scale would translate to a 0.10 [−0.01, 0.18] increase in affect and 0.18 [0.08, 0.32] unit increase in life satisfaction.

Second, extrinsic motivation impacted affect and life satisfaction negatively, such that a one-unit increase in extrinsic motivations were associated with a −0.09 [−0.21, 0.04] unit change in affect, and −0.11 [−0.28, 0.05] unit change in life satisfaction (figure 6, bottom row). Similar reciprocal effects of affect and life satisfaction on subsequent intrinsic and extrinsic motivation were also in evidence (see ESM). These parameters indicated smaller but positive effects of well-being on subsequent intrinsic motivation and negative effects of well-being on extrinsic motivation. By contrast to the play-time results, these analyses provided consistent evidence that the motivational quality of video game play was reliably linked to player well-being. Play experienced as a volitional experience was positively predictive of subsequent well-being whereas the opposite was true of engagement characterized by a sense of compulsion.

### 3.2. Attrition

Because we observed significant attrition and changes in variables over time, we took steps to assess the robustness of our conclusions to different assumptions about dropout. First, we found that individuals who dropped out were, on average, younger and had fewer years of game-play experience, reported greater levels of extrinsic motivations and lower levels of life satisfaction (but not affect), and played less than those who stayed for all three waves (see ESM). To specifically understand the robustness of our conclusions about game play and well-being, we then used multiple imputation to address four different hypothetical scenarios where data were missing not at random (MNAR) using the Δ-adjustment method [54]. First, we re-ran our analysis, focusing on the hours spent playing to affective well-being parameter, but assumed that individuals who did not respond would have reported Δ (−2, −1, 1, 2) lower/greater affective well-being (SPANE) compared to values imputed under a missing at random (MAR) model.

This analysis targeted the substantive hypothesis that individuals dropped out due to an unobserved decrease in well-being, which would threaten the validity of our main analyses that assumed a MAR missingness mechanism. Such an assumption would be in line with decreases in well-being and intrinsic motivation over time that we observed. We found that assuming negative Δs led to positive estimates of the cross-lagged parameter; suggesting that if dropout was associated with lesser well-being, game play would predict increased well-being (see ESM for details). Finally, when we assumed positive values of Δ, we found that estimates of the cross-lagged parameter became negative. Together, these findings suggest that our results, like any missing data analysis, are sensitive to unverifiable assumptions about the missingness mechanism. We nevertheless think that starting with a MAR assumption about missingness, as we have done, is a good starting point for this inquiry. Going forward, we need sampling strategies that rely less on the goodwill of players to participate. Such sampling will not make the assumptions about missingness verifiable, but they reduce the risk of systematic attrition.

## 4. Discussion

Evidence about video games' potential impacts so far has suffered from several limitations, most notably inaccurate measurement and a lack of explicit, testable causal models. We aimed to remedy these shortcomings by pairing objective behavioural data with self-reports of psychological states. Across six weeks, seven games and 38 935 players, our results suggest that the most pronounced hopes and fears surrounding video games may be unfounded: time spent playing video games had limited if any impact on well-being. Similarly, well-being had little to no effect on time spent playing.

We conclude the effects of playing are negligible because they are very unlikely to be large enough to be subjectively noticed. Anvari & Lakens [55] demonstrated that the smallest perceptible difference on PANAS, a scale similar to SPANE, was 0.20 (2%) on a 5-point Likert scale. In our study, 1 h day^−1^ increase in play resulted in 0.03 unit increase in well-being: assuming linearity and equidistant response categories, the average player would have to play 10 h more *per day* than typical to notice changes (i.e. 2% [0.26 units]) in well-being. Moreover, our model indicated 99% probability that the effect of increasing daily play time by one hour on well-being is too small to be subjectively noticeable. Even if effects steadily accumulated over time—an unrealistic assumption—players would notice a difference only after 17 weeks.

We also studied the roles of motivational experiences during play. Conceptually replicating previous cross-sectional findings [21], our results suggested that intrinsic motivation positively and extrinsic motivation negatively affects well-being. Motivations’ suggested effects were larger, and we can be more confident in them, than those of play time. However, the effect of a 1-point deviation from a player's typical intrinsic motivation on affect did not reach the threshold of being subjectively noticeable (0.10 estimate versus 0.26 threshold). Similarly: we cannot be certain a 1-point increase is a large or a small shift—participants' average range on the 7-point intrinsic motivation scale was 0.36. Until future work determines what constitutes an adequate ‘treatment’, these conclusions remain open to future investigation and interpretation. Our findings, therefore, suggest that amount of play does not, on balance, undermine well-being. Instead, our results align with a perspective that the motivational experiences during play may influence well-being [23]. Simply put, the subjective qualities of play may be more important than its quantity. The extent to which this effect generalizes or is practically significant remains an open question.

### 4.1. Limitations

Although we studied the play and well-being of thousands of people across diverse games, our study barely scratched the surface of video game play more broadly. Hundreds of millions of players play tens of thousands of games on online platforms. We were only able to study seven games, and thus the generalizability of our findings is limited [29]. To truly understand why people play and to what effect, we need to study a broader variety of games, genres and players. Moreover, we analysed total game time, which is the broadest possible measure of play. Although it is necessary to begin at a broad level [12,56], future work must account for the situations, motivations and contexts in which people play [57]. Additionally, play time is a skewed variable because a minority of players spend a great amount of time playing. This means that the Gaussian assumptions of the RICLPM might be threatened, and future simulation work should investigate how the RICLPM deals with skewed data. We also emphasize that our conclusions regarding the causal nature of the observed associations are tentative: without theoretical and empirical identification of confounds, our and future studies will probably produce biased estimates. Finally, industry-provided behavioural data have their own measurement error and there are differences between publishers. Independent researchers must continue working with industry to better understand behavioural data and their limitations.

## 5. Conclusion

Policymakers, healthcare professionals and game developers urgently need to know if video games influence players' well-being. We provided evidence on the causal impacts of play on well-being using objectively logged game-play behaviour. Our results show that the impact of time spent playing video games on well-being is probably too small to be subjectively noticeable and not credibly different from zero. Going forward, it is essential to cast a wider and deeper empirical and theoretical net and focus on the qualities of play experiences, in-game events, and players for whom effects may vary. Until then, limiting or promoting play based on time alone appears to bear neither benefit nor harm.

## Data Availability

The data, annotated code required to process and analyse them, supplementary analyses, and survey materials are available at https://osf.io/fb38n/. That page also includes details on data processing such as how session durations were cleaned, exact item wordings, complete surveys and additional data not analysed here. This study was not preregistered.
